# The Alvarado score for predicting acute appendicitis: a systematic review

**DOI:** 10.1186/1741-7015-9-139

**Published:** 2011-12-28

**Authors:** Robert Ohle, Fran O'Reilly, Kirsty K O'Brien, Tom Fahey, Borislav D Dimitrov

**Affiliations:** 1 HRB Centre for Primary Care Research, Division of Population Health Sciences, Royal College of Surgeons in Ireland, 123 St. Stephen's Green, Dublin 2, Ireland

## Abstract

**Background:**

The Alvarado score can be used to stratify patients with symptoms of suspected appendicitis; the validity of the score in certain patient groups and at different cut points is still unclear. The aim of this study was to assess the discrimination (diagnostic accuracy) and calibration performance of the Alvarado score.

**Methods:**

A systematic search of validation studies in Medline, Embase, DARE and The Cochrane library was performed up to April 2011. We assessed the diagnostic accuracy of the score at the two cut-off points: score of 5 (1 to 4 vs. 5 to 10) and score of 7 (1 to 6 vs. 7 to 10). Calibration was analysed across low (1 to 4), intermediate (5 to 6) and high (7 to 10) risk strata. The analysis focused on three sub-groups: men, women and children.

**Results:**

Forty-two studies were included in the review. In terms of diagnostic accuracy, the cut-point of 5 was good at 'ruling out' admission for appendicitis (sensitivity 99% overall, 96% men, 99% woman, 99% children). At the cut-point of 7, recommended for 'ruling in' appendicitis and progression to surgery, the score performed poorly in each subgroup (specificity overall 81%, men 57%, woman 73%, children 76%). The Alvarado score is well calibrated in men across all risk strata (low RR 1.06, 95% CI 0.87 to 1.28; intermediate 1.09, 0.86 to 1.37 and high 1.02, 0.97 to 1.08). The score over-predicts the probability of appendicitis in children in the intermediate and high risk groups and in women across all risk strata.

**Conclusions:**

The Alvarado score is a useful diagnostic 'rule out' score at a cut point of 5 for all patient groups. The score is well calibrated in men, inconsistent in children and over-predicts the probability of appendicitis in women across all strata of risk.

## Background

Acute appendicitis is the most common cause of an acute abdomen requiring surgery, with a lifetime risk of about 7% [1]. Symptoms of appendicitis overlap with a number of other conditions making diagnosis a challenge, particularly at an early stage of presentation [2]. Patients may be suitably triaged into alternative management strategies: reassurance, pursuit of an alternative diagnosis or observation/admission to hospital. If admitted to hospital, appropriate imaging may be required prior to proceeding to an appendectomy [3].

**Figure 1:**
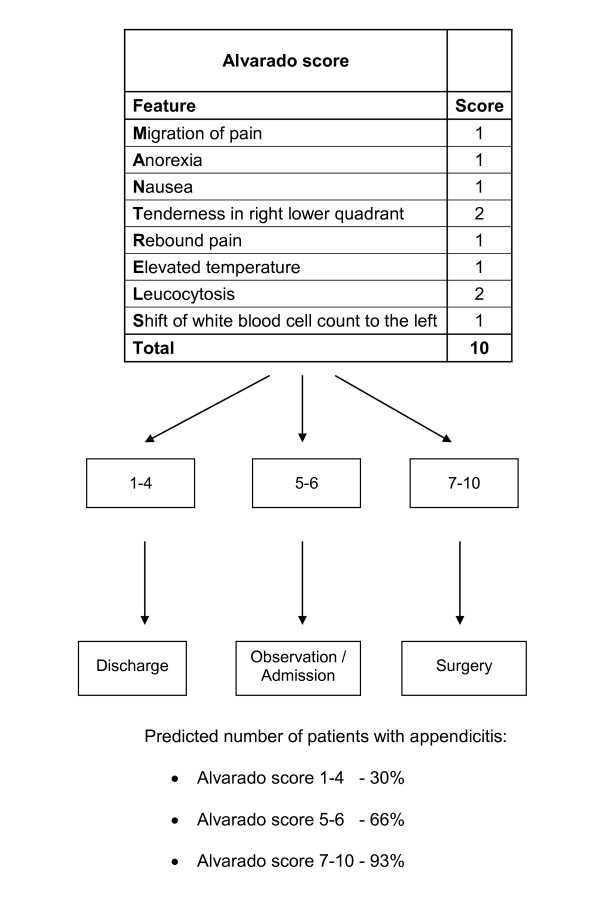
**Probability of appendicitis by the Alvarado score **[5]: **risk strata and subsequent clinical management strategy**.

Clinical prediction rules (CPRs) quantify the diagnosis of a target disorder based on findings of key symptoms, signs and available diagnostic tests, thus having an independent diagnostic or prognostic value [4]. They can also extend into clinical decision making if probability estimates are linked to management recommendations, and are subsequently referred to as clinical decision rules. CPRs have the potential to reduce diagnostic error, increase quality and enhance appropriate patient care [4]. In 1986, Alvarado constructed a 10-point clinical scoring system, also known by the acronym MANTRELS, for the diagnosis of acute appendicitis as based on symptoms, signs and diagnostic tests in patients presenting with suspected acute appendicitis (Figure 1) [5].

The Alvarado score enables risk stratification in patients presenting with abdominal pain, linking the probability of appendicitis to recommendations regarding discharge, observation or surgical intervention [5]. Further investigations, such as ultrasound and computed tomography (CT) scanning, are recommended when probability of appendicitis is in the intermediate range [6]. However, the time lag, high costs and variable availability of imaging procedures mean that the Alvarado score may be a valuable diagnostic aid when appendicitis is suspected to be the underlying cause of an acute abdomen, particularly in low-resource countries, where imaging is not an option.

A recent clinical policy document from the American College of Emergency Physicians reviews the value of using clinical findings to guide decision making in acute appendicitis [7]. Under the heading of the Alvarado score, they state that 'combining various signs and symptoms into a scoring system may be more useful in predicting the presence or absence of appendicitis'. Although not a strong recommendation, the Alvarado score is the only scoring system presented in the document.

The Alvarado score was originally designed more than two decades ago as a diagnostic score; however, its performance and appropriateness for routine clinical use is still unclear. The aim of this study was to perform a systematic review and meta-analysis of validation studies that assess the Alvarado score in order to determine its performance (diagnostic accuracy or discrimination at two cut-points commonly used for decision making, and calibration of the score). As studies have suggested that the accuracy of the Alvarado is affected by gender and age [8-12], we focused our analysis on three separate groups of patients: men, women and children.

## Methods

### Data sources and search strategy

**Figure 2:**
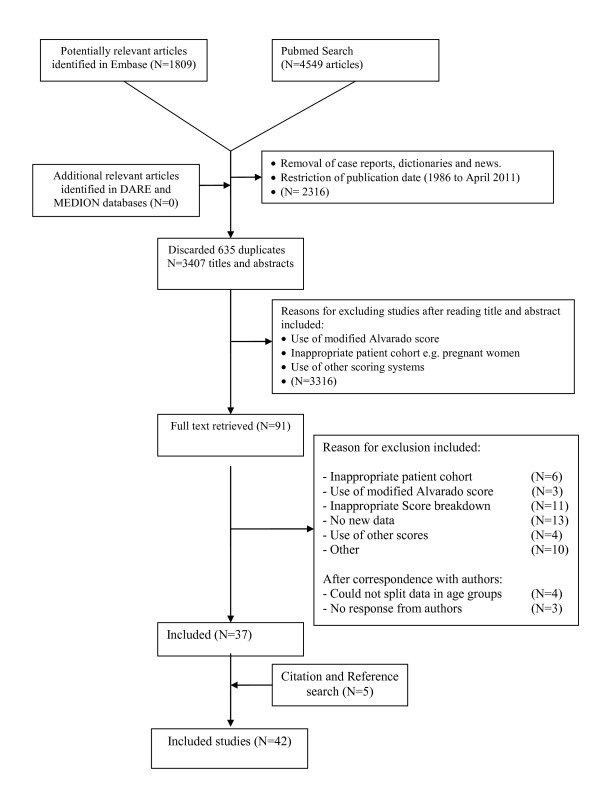
**Flow diagram for the selection of studies for inclusion in the meta-analysis**.

An electronic search was performed on PubMed (January 1986 to 4 April 2011), EMBASE (January 1986 to 4 April 2011), Cochrane library, MEDION and DARE databases. The search strategy is presented as a flow diagram in Figure 2. A combination of keywords and MeSH terms were used; 'appendicitis' OR 'alvarado' OR, 'Mantrels', was used in combination with 26 specific terms for CPRs, including 'risk score', 'decision rule', 'predictive value', 'diagnostic score', and 'diagnostic rule' [13]. A citation search of included articles was undertaken using Google Scholar. The references of included studies were also hand searched for relevant papers. Authors of recent papers (2001 onwards) were contacted when included studies did not report sufficient data to enable inclusion. No language restrictions were placed on the searches.

### Study selection

To be included in this study, participants had to be recruited from an emergency department or a surgical ward and present with symptoms suggestive of acute appendicitis, including abdominal pain, rebound tenderness, nausea, vomiting or elevated temperature. Each included study assessed the performance of the Alvarado score in comparison with the histological examination of the appendix following surgery (reference standard). For those who did not undergo appendectomy and histological examination, outpatient follow-up or no repeat presentation were used as alternative outcome measures. To be included, studies had to report results in a manner that allowed data to be extracted for either the diagnostic test accuracy analysis of the Alvarado score at specific cut points or the calibration analysis. Studies that focused on pregnant patients were excluded.

Two reviewers (RO and FO'R) completed the review process. The inclusion criteria were defined *a priori*. They reviewed titles and abstracts independently and after discussion decided which articles should be reviewed in full. Full text articles were reviewed independently by the same reviewers and any disagreements were resolved by discussion.

### Quality assessment, data extraction and statistical analysis

**Figure 3:**
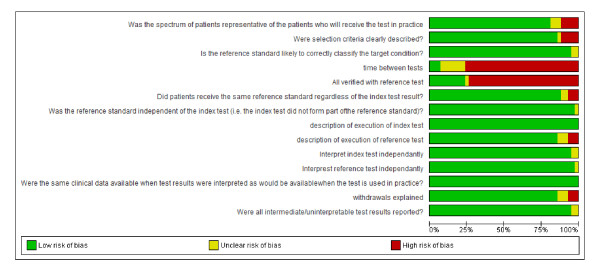
**Summary of quality assessment of included studies**.

Quality assessment of included papers was assessed using QUADAS (quality assessment of studies of diagnostic accuracy included in systematic reviews) and the risk of bias table in Review Manager 5 software from the Cochrane collaboration [14,15]. A summary of the quality of included papers is presented in Figure 3. Quality assessment was performed independently by two investigators (RO an FO'R) and any disagreements were resolved by discussion with a third investigator (KO'B).

#### Diagnostic accuracy of the Alvarado score

For the diagnostic accuracy (discrimination performance) of the Alvarado score, data were extracted and 2 × 2 tables constructed for use of the score as a criterion for admission (score 1 to 4 versus score 5 to 10, Figure 1) and as a criterion for surgery (score 7 to 10 versus score 1 to 6, Figure 1). Data extraction was carried out independently by two reviewers (RO and FO'R) and the data compared. A bivariate random-effects model was used to compute summary diagnostic sensitivity and specificity which allowed for heterogeneity beyond chance as a result of clinical and methodological differences between the studies to be taken into account. Heterogeneity was assessed using the variance of logit transformed sensitivity and specificity, where smaller values indicate less heterogeneity across studies. HSROC (hierarchical summary receiver operating characteristic) curves were also constructed with 95% confidence regions illustrating the precision with which pooled values are estimated and a 95% prediction region, illustrating the amount of between-study variation. Analyses were carried out using STATA software (StataCorp LP, College Station, TX, 77845, USA), using the "metandi" command [16,17].

#### Calibration analysis of the Alvarado score

The initial derivation study of the Alvarado score was used as the predictive model against which all validation studies were compared [5]. The number of patients diagnosed with appendicitis as estimated by the Alvarado score (predicted events) was compared to the actual number of patients with appendicitis (observed events) in each of the validation studies. The analysis was performed separately across three risk strata of the Alvarado score (low risk, score 1 to 4; intermediate risk, score 5 to 6; and high risk, score 7 to 10) (Figure 1). Within each risk stratum, each of the three main study populations, men, women and children were analysed separately [8,10-12,18].

The results from the calibration assessment were presented as risk ratios (RRs with 95% confidence intervals) and are illustrated as forest plots. RR < 1.00 indicates an under-prediction of appendicitis by the score (observed number with appendicitis is greater than the predicted number) and RR > 1.00 indicates an over-prediction of appendicitis by the score (observed number with appendicitis is less than the predicted number). RR = 1 indicates a matched calibration between observed and predicted numbers. Review Manager 5 software from the Cochrane collaboration was used to perform the pooled analysis, determine heterogeneity and produce the forest plots. RRs with their 95% CIs were computed by the Mantel-Haenszel (M-H) method. A random-effects model was used and heterogeneity assessed by *I^2 ^*statistic.

Prevalence was investigated as a source of heterogeneity in a subgroup analysis. Studies were dichotomised, based on their prevalence, as being either higher or lower than the Alvarado's derivation study; the effect on heterogeneity and the calibration of the score were also investigated.

## Results

The literature search yielded > 3,000 titles and abstracts for screening. The full text of 91 articles met the eligibility criteria, and these articles were retrieved (Figure 2). Thirty-seven articles were included from the search, and a further five articles were retrieved after citation searching, with a total of 42 articles meeting the inclusion criteria. The included studies came from a variety of settings and countries (Table 1). Nine studies took place in a surgical ward; three studies only specified that patients were hospitalised, all remaining studies were performed in an emergency department setting. Detailed characteristics of all included studies are presented in Table 1.

**Table 1:** Characteristics of included studies

First author, study year [reference]	Number of patients	Age (years)	Gender n	Appendicitis prevalence (%)	Country	Setting	Study type	Patient population
Abdeldaim 2007 [19]	242	Median 42 Range 8 to 76	Male 90 Female 152	51	Ireland	Emergency department	Retrospective	Right iliac fossa pain
Al Qahtani 2004 [8]	211	Mean 32 Range 13 to 70	Male 125 Female 86	57	Saudi Arabia	Emergency department	Prospective	Suspected acute appendicitis
Alvarado 1986 [5]	277	Mean 25.3 Range 4 to 80	Male 131 Female 96	82	USA	Hospital inpatients	Prospective	Abdominal pain
								
Arain 2001 [20]	100	Mean 19.9	Males 44 Females 56	48	Pakistan	Surgical unit	Prospective	Suspected acute appendicitis
Baidya 2007 [44]	231	Mean 26.3 Range 16 to 65	Male 141 Female 90	52	India	Emergency department	Prospective	Right *iliac fossa *pain
Bond 1990 [21]	187	Range 0 to 18		61	USA	Emergency department	Prospective	Abdominal pain
Borges 2003 [22]	76	Age 2 to 6 = 23 Age 7 to 10 = 38 Age > 10 = 15	Male 40 Female 36	71	Brazil	Emergency department	Prospective	Suspected acute appendicitis
Canavosso 2008 [23]	224	Mean 26.65 Range 13 to 82	Male 117 Female 10	84	Argentina	Emergency department	Prospective	Right lower quadrant pain
Chan 2001 [25]	148	Mean 29 Range 10 to 73	Male 107 Female 41	34	Singapore	Emergency department	Retrospective	Suspected acute appendicitis
Chan 2003 [24]	175	Mean 30 Range 8 to 73	Male 130 Female 45	43	Singapore	Emergency department	Prospective	Right iliac fossa pain
Denizbasi 2003 [45]	221	Mean 26.6	Male 112 Female 109	79	Turkey	Emergency department	Prospective	Abdominal pain and suspected acute appendicitis
Escriba 2011 [42]	99	Mean 11.2 Range 4 to 17.8	Male 62 Female 37	42	Spain	Emergency department	Prospective	Abdominal pain/suspected appendicitis
Farahnak 2007 [26]	21	-	-	48	Iran	Emergency department	Prospective	Abdominal pain
Gwynn 2001 [27]	215	-	-	66	USA	Emergency department	Retrospective	Abdominal pain
Hsiao 2005 [28]	222	Mean 9.4 Range 1 to 13	Male 146 Female 76	50	Taiwan	Emergency department	Retrospective	Suspected acute appendicitis
Kang 1989 [46]	62	Mean 45.8 Range 18 to 78	Male 42 Female	68	China	Hospital inpatients	Prospective	Suspected acute appendicitis
Khan 2005 [29]	100	Mean 20.2 Range 9 to 56	Female 59 Male 41	54	Pakistan	Surgical ward	Prospective	Suspected acute appendicitis
Kim 2006 [9]	211	-	-	83	Korea	Surgical ward	Retrospective	Suspected acute appendicitis
Kim 2008 [18]	157	Mean 37.1 Range 15 to 84	-	57	Korea	Emergency department	Prospective observational study	Abdominal pain
Lada 2005 [10]	83	Mean 27.5 Range 15 to 75	Male 43 Female 40	88	Argentina	Emergency department	Prospective	Suspected acute appendicitis
Malik 2000 [56]	100	Mean 22 Range 14 to 18	Male 81 Female 19	92	Pakistan	Surgical unit	Prospective	Suspected acute appendicitis
McKay 2007 [31]	150	Mean 34 Range 18 to 76	Male 78 Female 65	32	USA	Emergency department	Retrospective	Abdominal pain
Memon 2009 [32]	100	Mean age 24 Range 13 to 55	Male 65 Female 35	91	Pakistan	Surgical Ward	Prospective study	Suspected acute appendicitis
Muenzer 2010 [47]	28	Test Cohort Mean 11 Validation cohort Mean 11 Range 2 to 17	Test cohort Male 10 Female 10 Validation cohort Male 4 Female 4	54	USA	Emergency department	Unclear	Abdominal pain
Owen 1992 [11]	215		Male 75 Female 70 Children 70	58	Wales	Emergency department	Prospective observational study	GP referral for Suspected acute appendicitis
Petrosyan 2008 [33]	1,630	Male: Median 29 yrs Range 3 to 85 yrs Female: Median 34 yrs Range 2 to 86 yrs	Male 928 Female 702	54	USA	Emergency department	Retrospective	Right lower quadrant pain and suspected acute appendicitis
								
Rezak 2011 [40]	59	Mean 8.5 Range 3 to 16	Male 43 Female 16	51	USA	Community teaching hospital	Retrospective	Suspected appendicitis
Saidi 2000 [43]	128		Male 49 Female 79	35	Iran	Emergency department	Prospective	Suspected acute appendicitis
Sanabria 2007 [34]	374	Mean 29.5 Range 15 to 71	Male 178 Female 196	55	Columbia	Emergency department	Prospective	Right iliac fossa pain and suspected acute appendicitis
Schneider 2007 [12]	588	Median 11.9 Range 3 to 21		33	USA	Emergency department	Prospective cohort	Suspected acute appendicitis
Shreef 2010 [41]	350	Mean 9.3 Range 8 to 14	Male 228 Female 122	38	Egypt, Saudi Arabia	Emergency department	Prospective	Suspected acute appendicitis
Shrivastava 2004 [57]	100		Male 45 Female 30	78	India	Emergency department	Prospective	Suspected acute appendicitis
Singh 2008 [35]	100	Mean 22.6 Median 25	Male 55 Female 45	62	India	Surgical Ward	Prospective	Suspected acute appendicitis
Soomro 2008 [36]	227	Mean 20.47 Range 10 to 62	Male 150 Females 77	55	Pakistan	Emergency department	Prospective	Suspected acute appendicitis
Stephens 1999 [37]	94	Mean 44 Range 3 to 79	Males 46 Female 48	89	USA	Surgical unit	Retrospective	All patients who underwent appendectomy for suspected acute appendicitis
								
Tade 2007 [38]	100	Range 17 to 56	Males 63 Female 37	34	Nigeria	Emergency department	Prospective	Right *iliac fossa *pain and suspected acute appendicitis
Wani 2007 [30]	96	Mean 25.46 Range 7 to 70	Male 48 Female 48	70	India	Surgical unit	Prospective	Suspected acute appendicitis
Yildirim 2008 [39]	143	Mean 34 Range 18 to 76	Male 78 Females 65	85	Turkey	Emergency department	Prospective study	Abdominal pain
Winn 2004 [58]	142			39	Australia	Surgical ward	Retrospective	Suspected acute appendicitis
								
Subotic 2008 [59]	57	Mean 27.5 Range 16 to 70	Male 27 Female 30	84	Serbia	Emergency department	Prospective	Suspected acute appendicitis
Andersson 2008 [60]	229	Mean 23	Males 105 Females 124	33	Sweden	Emergency department	Prospective	Suspected acute appendicitis
Prabhudesai 2008 [61]	60	Mean 25.4	Male 27 Female 33	40	UK	Emergency department	Prospective	Suspected acute appendicitis

Results of the quality assessment are shown in Figure 3. The overall quality of the included studies is considered acceptable for most of the quality items. The assessment of the clinical variables composing the Alvarado score and the reference standard for diagnosis (histological results of the appendectomy) were interpreted independently in most studies. The retrospective studies rarely reported if the scorer was aware of the final diagnosis (blind assessment). The quality item, 'time between tests', is the time between administering the Alvarado score and verifying the diagnosis with pathology or follow-up and was very poorly reported. As part of our inclusion criteria, all studies had to confirm the diagnosis of appendicitis on those undergoing appendectomy; however, follow-up of those discharged was poor in the majority of studies (item 'All verified with reference test').

### Diagnostic accuracy of the Alvarado score

The Alvarado score discriminated well as an observation/admission criterion (cut point of 5) by achieving high pooled sensitivity of 99% overall (n = 28 studies, [5,8,10,18-42]) and in studies where data were available, it also performed well in the subgroup analysis for men, woman and children (pooled sensitivities: 0.96 for men, n = 5 [23,30,33-35]; 0.99 for women, n = 5 [23,30,34,35,43] and 0.99 for children, n = 9 [10,21,23,27,28,30,40-42]) (Table 2 and Additional file 1 - Figure S1). In patients presenting with higher Alvarado scores (cut point of 7, the criterion for surgery), pooled diagnostic accuracy results had more limited clinical value (pooled specificity for all studies 0.82, n = 29, [5,8,10,11,18-25,27-32,34-38,41,42,44-47]), with pooled specificities ranging from 0.57 for subgroup analysis of men (n = 6, [9,23,30,34,35,45]), 0.73 for subgroup analysis of women (n = 7, [9,23,30,33-35,45]) and 0.76 for subgroup analysis of children (n = 9, [10,21,23,27,28,30,41,42,47]) (Table 2 and Additional file 1 - Figure S1).

**Table 2:** Summary estimates of sensitivity and specificity calculated by a bivariate random-effects model

	Studies	n	Sensitivity (95% CI)	Variance logit (sensitivity)	Specificity (95% CI)	Variance logit (specificity)
Observation/ Admission (Cut point 5)	All studies	28	0.99 (0.97 to 0.99)	3.37	0.43 (0.36 to 0.51)	0.61
	Men	5	0.96 (0.88 to 0.99)	1.09	0.34 (0.24 to 0.47)	0.06
	Women	5	0.99 (0.92 to 0.99)	2.12	0.35 (0.14 to 0.64)	1.51
	Children*	9	0.99 (0.83 to 1.00)	8.99	0.57 (0.41 to 0.72)	0.79
Surgery (Cut point 7)	All studies	29	0.82 (0.76 to 0.86)	0.48	0.81 (0.76 to 0.85)	0.46
	Men	6	0.88 (0.75 to 0.95)	1.15	0.57 (0.40 to 0.73)	0.44
	Women	7	0.86 (0.78 to 0.92)	0.44	0.73 (0.58 to 0.84)	0.62
	Children*	9	0.87 (0.76 to 0.93)	0.98	0.76 (0.55 to 0.89)	1.50

* For the purpose of this study Children are defined as any participant under the age of 18 years of age.

Overall, heterogeneity was high when all studies were included and was particularly high in the children subgroup as indicated by the variance logit transformed sensitivity and specificity (Table 2) and the prediction ellipses on the SROC curves Additional file 1 - Figure S1).

### Calibration of the Alvarado score

**Figure 4:**
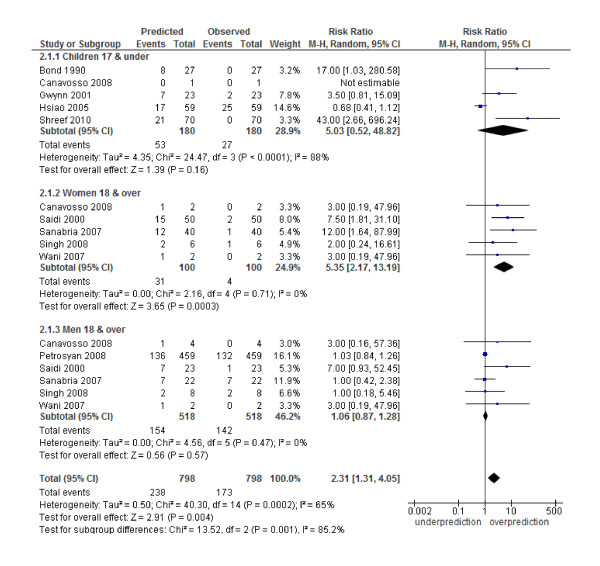
**Low risk group (1 to 4): predicted versus observed cases with appendicitis in children, women and men**.

**Figure 5:**
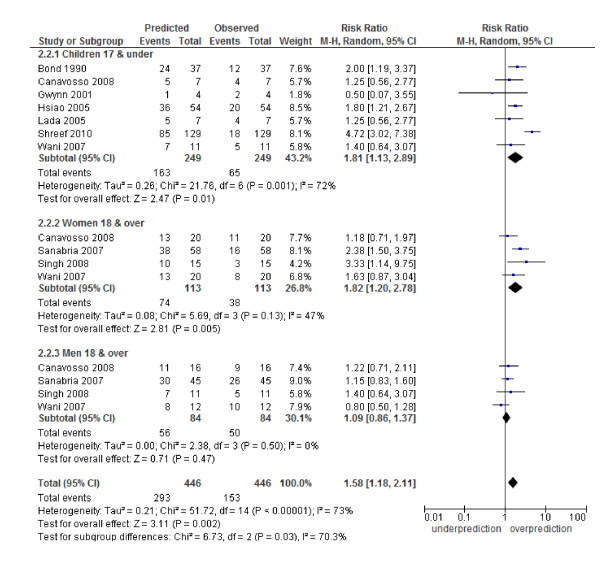
**Intermediate risk group (5 to 6): predicted versus observed cases with appendicitis in children, women and men**.

**Figure 6:**
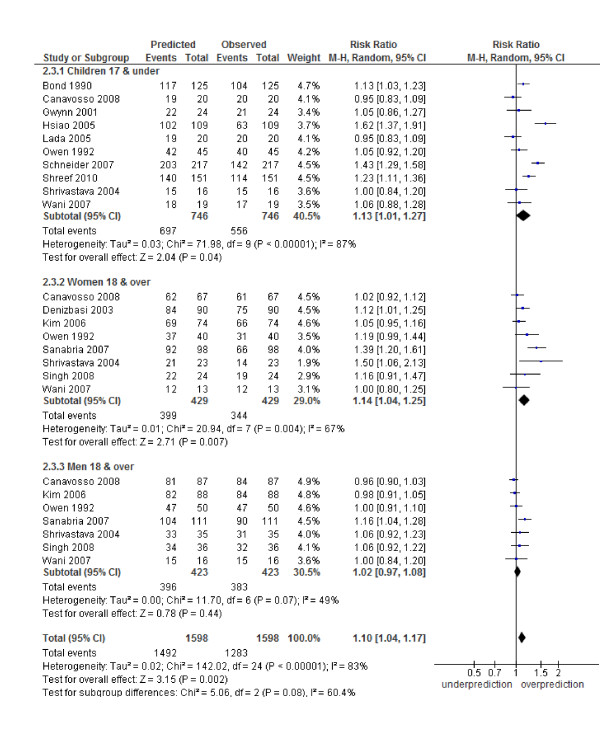
**High risk group (7 to 10): predicted versus observed cases with appendicitis in children, women and men**.

The Alvarado score performed well in all three risk strata for men: (low risk RR 1.06, 95% CI 0.87 to 1.28; intermediate risk 1.09, 0.86 to 1.37 and high risk 1.02, 0.97 to 1.08). In women, there was a systematic over-prediction across all risk strata: low risk (RR 5.35, 2.17 to 13.19), intermediate risk (RR 1.82, 1.20 to 2.78) and high risk (RR 1.14, 1.04 to 1.25). In children, there was a non-significant trend towards over-prediction in the low risk strata (5.03, 0.52 to 48.82) and a significant over-prediction in the intermediate risk category (1.81, 1.13 to 2.89) and high risk strata (1.13, 1.01 to 1.27) (Figures 4, 5, 6). Heterogeneity in terms of between-study predicted/observed risk ratio estimates is apparent in children across all risk strata and in women at a high risk (*I^2 ^*> 50%), and, therefore, these pooled estimates should be treated with caution.

In a subgroup analysis based on prevalence (Additional file 1 - Figure S2), the high prevalence category consisted of six studies [9,10,23,32,37,39] - the score predicted well in this group and heterogeneity was below 50% in the high and low risk groups (low risk RR 0.65, 95% CI 0.25 to 1.75, *I^2 ^*= 34%; intermediate risk RR 0.99, 95% CI 0.70 to 1.40, *I^2 ^*= 72%; high risk RR 0.99, 95% CI 0.96 to 1.02, *I^2 ^*= 0%). The low prevalence subgroup consisted of 24 studies, there was a significant overprediction across all risk strata; however, heterogeneity was extremely high (*I^2 ^*= 78% to 85%) suggesting that other factors, perhaps age and gender, contributed to the high levels of heterogeneity in this group. Unfortunately, not enough studies had age and gender information to allow us to do further subgroup analysis.

## Discussion

### Principal findings

This systematic review shows that the Alvarado score at the cut point of 5 performs well as a "rule out" CPR in all patient groups with suspected appendicitis. Pooled diagnostic accuracy in terms of "ruling in" appendicitis at a cut-point of seven points is not sufficiently specific in any patient group to proceed directly to surgery. In terms of calibration, the observed, predicted estimates in men suggest the score is well calibrated across all risk strata. Application of the Alvarado score in women over-predicts the probability of appendicitis across all strata of risk and should be used with caution. The validity of the Alvarado score in children was inconclusive; the calibration analysis showed high levels of heterogeneity across all risk strata. Further validation studies are required before clinical implementation of the Alvarado score for this age group could be recommended.

### Clinical implications

A recent clinical policy document from the American College of Emergency Physicians reviewed the value of using clinical findings to guide decision making in acute appendicitis [7]. They state that combining various signs and symptoms, as in the Alvarado score, may be more useful in predicting the presence or absence of appendicitis. This systematic review supports the use of the Alvarado score as a triage CPR that can be applied to 'rule out' appendicitis at a score below five points (sensitivity 94% to 99%), but not as a 'rule in' for appendicitis. Patients with a score less than 5 can be considered for discharge with the proviso that watchful waiting and re-assessment may be required if symptoms change or deteriorate. The advantage of applying the Alvarado score in this way is that resources in terms of admitting a patient to hospital or performing diagnostic imaging can be reserved for higher-risk scoring patients. Such an approach may be particularly useful in low-resource settings where diagnostic testing is limited or not available [38].

Based on the results of this review, the Alvarado score at a cut-off of five points compares favourably with other CPRs used in clinical practice. The Ottawa ankle and knee rules represent "rule out" CPRs of similarly high sensitivity that are used in emergency departments to decide if a patient should be referred for radiography to determine if their ankle or knee is fractured. The application of these CPRs is to identify those patients with a very low risk of fracture, where fracture can be confidently ruled out and the patient can be discharged without unnecessary imaging. For this purpose, it is important that such CPRs have high sensitivity. Meta-analysis of validation studies show these rules achieve high sensitivity that is comparable to the Alvarado score at a cut-off of five points (ankle rule - 97.6% [48], knee rule - 98.5% [49] and Alvarado score at cut-off of five points - 99%).

The use of the Alvarado score as a 'rule in' CPR for surgery at a cut point of 7 is not supported by our diagnostic test accuracy results. Our analysis indicates that the Alvarado score has moderate to high sensitivity (all studies 82%, men 88%, women 86% and children 87%) and a moderate specificity (all studies 81%, men 57%, women 73% and children 76%), suggesting it is not sufficiently accurate to rule in or rule out surgery (Table 2). However, several studies report that the application of Alvarado score as a sole decision criterion for surgery (cut point of 7) produces negative appendectomy rates of 13.3%, 15.6%, 16.2% and 14.3%, respectively, without an increase in perforations [11,20,29,35]. This is comparable with a clinician's judgment in other reports (17.1%, 12%, 12.5% and 11%) [5,8,19,27]. An Alvarado score ≥ 7 is useful at identifying those at high risk of acute appendicitis who require a surgical consultation or further diagnostic imaging, it should not be used as the sole criterion for ruling in surgery in any patient group.

During the last 10 years, the diagnostic imaging by CT scan in the diagnosis of appendicitis has become a common practice. In some centres over 90% of the patients presenting with suspected appendicitis undergo CT imaging. CT has a high sensitivity and specificity for the diagnosis of appendicitis and it considerably reduces the level of negative appendectomy. However, some studies have shown that the use of CT does not necessarily change the clinical management of a patient, especially in those at high risk [33,50]. CT imaging may also delay the time of operation and, therefore, may increase the subsequent risk of perforation [51]. Assessing the use of the Alvarado score and CT imaging as a series of diagnostic investigations on all these types of outcomes is warranted.

Lastly, the results of this systematic review have important implications in low-resource countries. First, in low-resource settings where the decision to operate may be based on a clinical judgment, the Alvarado score provides an accurate and consistent triage tool for ruling out appendicitis and identifying those at higher risk who would benefit at most from an admission to a hospital. Second, the Alvarado score could serve as a simplified tool for the emergency physician in order to stratify patients for referral for surgical consultation.

### Context of other research

Although the Alvarado score was developed in a mixed gender population, the ratio of men to woman was 1.4:1 and the score has subsequently been shown to perform poorly when applied to women of child-bearing age [8-11]. It is also possible that a certain loss of diagnostic information may have occurred due to dichotomisation when the score was originally constructed in the derivation study. Abdominal pain in women is a diagnostically challenging symptom as there are more diagnostic possibilities aside from appendicitis, such as pelvic inflammatory disease and other gynaecological pathologies. Alternative risk scores or CPRs, such as Lindeberg [52], Eskelinen [53] and Fenyo [54] scores for appendicitis, have different numerical values for symptoms depending on whether the patient is male or female [55]. The Van Way, Teicher and Arnbjornssion scores include gender as one of their components [55]. Of note, Ohman *et al*. [55] reported that the Alvarado score outperformed each of these other scores.

Distinguishing appendicitis from other causes of abdominal pain in children is also challenging, particularly in young children who cannot articulate how they feel or where the pain is. There is also a wide variation in presenting symptoms and it is often hard to elicit the classical presentation [2]. The use of symptoms and signs to identify children who are at risk of acute appendicitis is particularly appealing as diagnostic imaging using a CT scan exposes children to ionizing radiation and the diagnostic accuracy of ultrasound is still uncertain [7]. A recent review found that "fever" in a child is the single most important sign associated with appendicitis, followed by rebound tenderness and migration of pain, suggesting that the Alvarado score may not be the most appropriate scoring system for children as double points are scored for tenderness in the right lower quadrant and leukocytosis, but only one point for each of all other signs (Figure 1) [2]. This review also reported the accuracy of clinical scoring systems, including the Alvarado score, where the likelihood ratio for cut points of 7 and 5 (based on three studies) was similar to our pooled estimates (cut point of 7, 3.1 and 3.5; cut point of 5, 0.05 and 0.02, respectively). Two of the studies in the review by Bundy *et al*. were included in this review [11,21].

### Strengths and weaknesses of the present study

Our study does have a number of limitations. First, although it is usually related mainly to discrimination, some degree of misclassification may have also occurred when calibration was considered by comparing predicted versus observed patients with appendicitis; however, given the high levels of diagnostic performance, overall (especially, at the cut-off point of 5) this appears unlikely.

Second, a moderate to high level of heterogeneity was shown across the included studies in both the diagnostic test accuracy analysis and the calibration analysis (Table 2, Figures 4, 5, 6 and Additional file 1 - Figure S1). There are a number of possible sources for heterogeneity, including chance; variation in pre-test probability; the case mix of men, woman and children; a threshold effect caused by observer variation in the measurement of signs and symptoms; no active follow-up of patients discharged and other unanticipated factors. We addressed a number of these potential sources of heterogeneity by performing subgroup analysis. The main focus of this paper was an analysis of such subgroups as men, woman and children. The performance of the score has been shown by others [8-12] to be affected by age and gender and, therefore, high heterogeneity in the overall results may be due to the gender and age spectrum of the patients in the included studies (for example, Table 2, all studies, variance logit sensitivity is 3.37). The prevalence of appendicitis among the validation studies was highly variable (range 32% to 91%, Table 1). Although this was investigated in a subgroup analysis a good deal of heterogeneity still existed, suggesting that other factors contributed towards heterogeneity in this analysis (Additional file 1 - Figure S2). Unfortunately it was not possible to do further subgroup analysis based on age, gender and prevalence due to a lack of studies with this information. Finally, a number of the studies used no repeat admission as a negative proxy measure for appendicitis. The lack of active follow-up in these studies may have led to misclassification if patients presented to a different hospital. This may have led to a lower reporting of appendicitis cases, particularly in the low-risk groups, and inflated our estimates of sensitivity and specificity.

Finally, although we used an up-to-date systematic search strategy, we acknowledge that it was not exhaustive and it is possible, as with all systematic reviews, that relevant articles may have been missed. As we did not search the grey literature, there is also the possibility of publication bias, with smaller negative studies being under reported, leading to inflated estimates of sensitivity and specificity in our meta-analysis.

### Future research and applications in clinical practice

The criteria for selection of the included articles were broad and reflected the nature of the validation studies themselves, producing a high level of heterogeneity across the studies in some of the risk strata. Further analyses are needed to explore the reasons behind the over-prediction of the Alvarado score in women. Such future analyses may suggest ways to adjust the predicted estimates according to the population prevalence in the various settings and/or a re-calibration or re-modelling of the score itself, mainly in low-prevalence settings and in women. Obtaining individual-level data from the validation studies to perform meta-analysis of the risk ratios can make such approaches possible, particularly for the more detailed exploration of the various sources of heterogeneity.

## Conclusions

This study shows that the Alvarado score accurately predicts appendicitis and is well calibrated in men. As a decision rule for observation/admission, the Alvarado score performs well as a 'rule out' criterion (high sensitivity). As a decision rule in relation to surgery the Alvarado score cannot be used to 'rule in' a diagnosis of appendicitis without surgical assessment and further diagnostic testing. Patients presenting in the emergency department and in primary care settings, especially in low-resource countries, could benefit from the implementation of the Alvarado score as a triage decision rule.

## Abbreviations

CPRs: clinical prediction rules; CT: computed tomography; HSROC: hierarchical summary receiver operating characteristic; MANTRELS, a popular **mnemonic **used to remember the Alvarado score factors: **M**igration to the right iliac fossa, **A**norexia, **N**ausea/Vomiting, **T**enderness in the right iliac fossa, **R**ebound pain, **E**levated temperature (fever), **L**eukocytosis, and **S**hift of leukocytes to the left; M-H method: Mantel-Haenszel method; QUADAS: quality assessment of studies of diagnostic accuracy included in systematic reviews; RR: risk ratio.

## Competing interests

The authors declare that they have no competing interests.

## Authors' contributions

RO, FO'R and KO'B were responsible for study protocol, data collection, and data elaboration and analysis. TF and BDD were responsible for the concept and study design and contributed to the data analysis and their interpretation, and drafting the report. All authors contributed to the interpretation of results, critical review of the manuscript and agreed on the final layout for submission. BDD is guarantor of the study.

## Pre-publication history

The pre-publication history for this paper can be accessed here:

http://www.biomedcentral.com/1741-7015/9/139/prepub

## Supplementary Material

Additional file 1 **Figure S1**. Summary ROC curves (sensitivity and specificity with 95% CIs are presented in Table 2). **Figure S2**. Predicted versus observed cases with appendicitis per study, sub-grouped by prevalence. The studies were re-grouped into high- or low-prevalence according to the prevalence cut-off point (82%) found in Alvarado's derivation study: A. Low risk, score 1 to 4; B. Intermediate risk, score 5 to 6; C. High risk, score 7 to 10. Click here for file
