# Supplementary material for: The Alvarado score for predicting acute appendicitis: a systematic review
Source: BMC Med. 2011 Dec 28;9:139. doi: 10.1186/1741-7015-9-139 (PMC3299622; doi:10.1186/1741-7015-9-139)

**Additional file**

**Appendix**

**Figure S1**: Summary ROC curves (sensitivity and specificity with 95% CIs are presented in Table 2)

Observation/Admission (Men) Observation/Admission (Women) Observation/Admission (Children)

Surgery (Men) Surgery (Women) Surgery (Children)

**Figure S2:** Predicted versus observed cases with appendicitis per study, sub-grouped by prevalence. The studies were re-grouped into high- or low-prevalence according to the prevalence cut-off point (82%) found in Alvarado’s derivation study.

1. Low risk, score 1-4


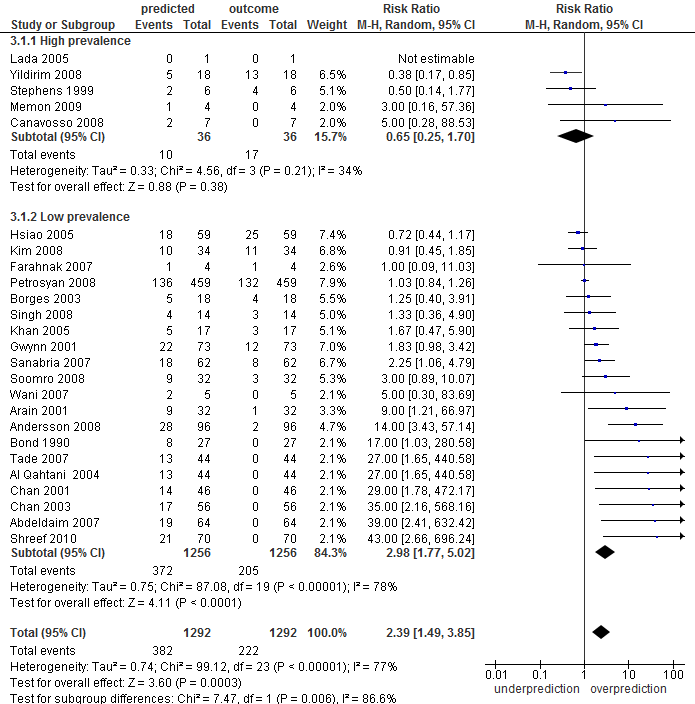


1. Intermediate risk, score 5-6


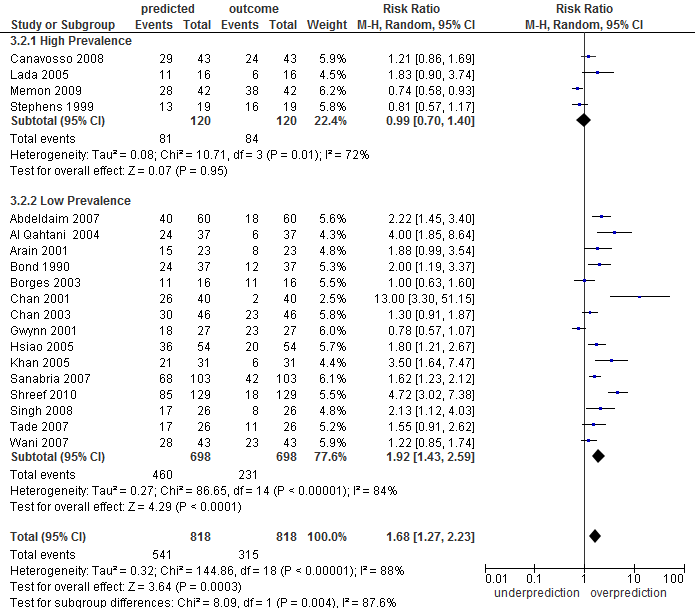


1. High risk, score 7-10


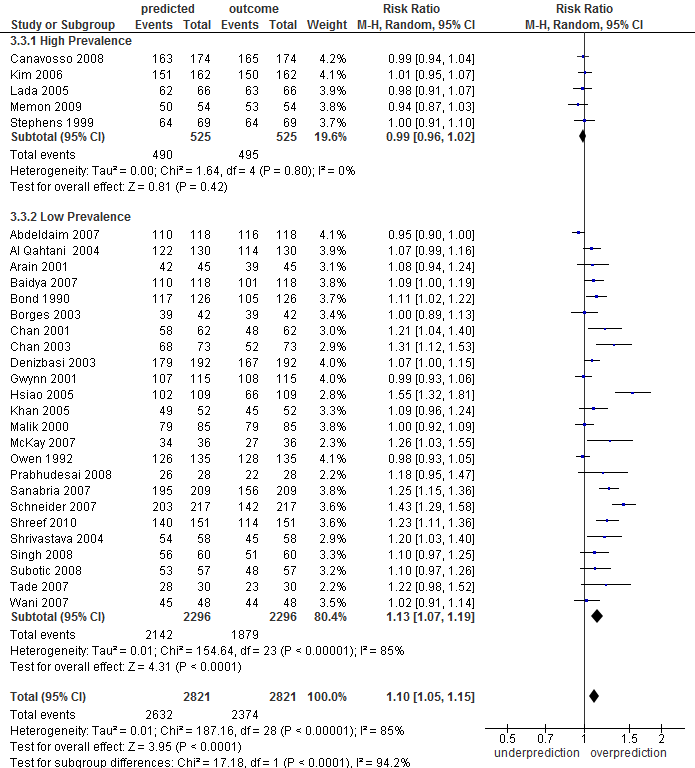

Supplement: Additional file 1 — Figure S1. Summary ROC curves (sensitivity and specificity with 95% CIs are presented in Table 2). Figure S2. Predicted versus observed cases with appendicitis per study, sub-grouped by prevalence. The studies were re-grouped into high- or low-prevalence according to the prevalence cut-off point (82%) found in Alvarado's derivation study: A. Low risk, score 1 to 4; B. Intermediate risk, score 5 to 6; C. High risk, score 7 to 10. [file 1741-7015-9-139-S1.DOC]
